# The influence of sustainable sport nutrition practices on youth sports participants

**DOI:** 10.3389/fnut.2026.1730987

**Published:** 2026-01-23

**Authors:** Jianfeng Deng

**Affiliations:** Guangzhou Songtian Polytechnic College, Guangzhou, China

**Keywords:** athletes, China, environmental awareness, health performance, sports participation, sustainable nutrition

## Abstract

**Background:**

Sustainable sports nutrition is increasingly recognized as a means of enhancing the athlete’s performance while reducing negative environmental impacts, however, empirical data in the context of adolescent athletes is limited.

**Objectives:**

This study examined sport nutrition knowledge, environmental awareness, and sustainable dietary behaviors among adolescent athletes, and their associated with motivation and sports participation.

**Methods:**

A mixed-methods design was used to examine sustainable nutrition behaviors and their motivational, contextual, and social determinants. A total of 312 athletes were invited to participate, and 120 completed a structured survey (response rate: 38%). Measures included a modified Sport Nutrition Knowledge Questionnaire, a validated environmental awareness subscale (*α* > 0.78), and a composite Sustainable Practice Score (SPS; α = 0.81).

**Results and discussion:**

Quantitative analyses showed that 78% of the participants reported moderate awareness of the environmental impact of food choices, and 62% had adopted at least one sustainable dietary practice. SPS was positively correlated with sports nutrition knowledge (r = 0.39, *p* < 0.001) and environmental awareness (r = 0.46, *p* < 0.001). Regression analyses indicated that sustainable nutrition practices significantly predicted motivation (*β* = 0.32, *p* < 0.01) and participation frequency (β = 0.29, *p* < 0.01), with environmental awareness moderating both relationships. Qualitative findings highlighted perceived physiological benefits, social influences, and structural barriers as a key theme influencing adoption.

**Conclusion:**

In general, adolescent athletes demonstrated developing and context-dependent sustainable dietary behaviors shaped by nutrition knowledge, environmental awareness, and socio-cultural influences. Enhancing sustainability-oriented educational interventions within youth sport settings may support both performance-related outcomes and the development of environmental responsibility.

## Graphical abstract

**Figure:**
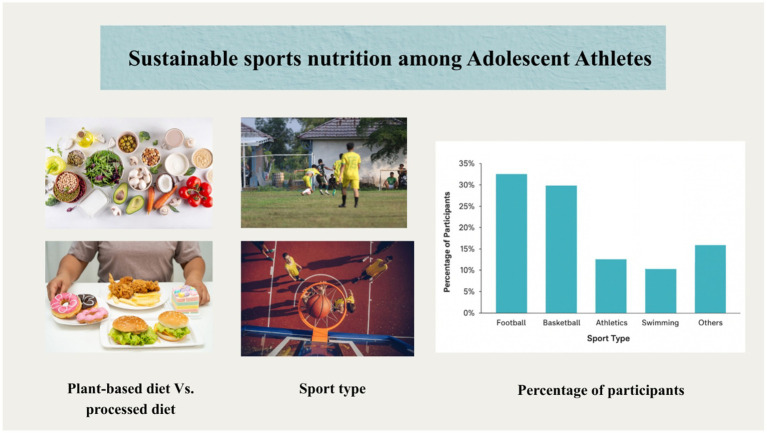

## 1 Introduction

Sustainable sports nutrition refers to nutritional practices aimed at maximizing athletic performance and recovery while simultaneously minimizing environmental effects. Nutritional status has both positive and negative impacts on the health and performance of athletes (1). This paper uses sustainability across three measurable domains namely: (1) source-related sustainability, which refers to a preference for locally grown, minimally processed, and predominantly plant-based foods; (2) resource efficiency, which implies the proper use of food, energy, and water resources by reducing reliance on high-carbon, water-intensive animal protein sources and minimizing single-use packaging; and (3) behavioral stewardship, which denotes deliberate actions by athletes to select, consume, and manage foods in ways that reduce food waste and promote ecological consciousness (2). The definition is based on a dual-focus perspective that contributes nutritional adequacy encompassing energy, protein, and micronutrient intake that supports athletic performance with environmental responsibility, which is the efficiency of resource-usage in terms of frequency and awareness indicators (3).

This group is of particular interest because adolescents are developing long-term dietary beheviors that often persist into adulthood (4). In addition, although many sports organizations promote social responsibility, the implementation of sustainable nutrition systems complements the broader educational framework by fostering accountability, resilience, and ethical responses to global challenges (5).

Previous studies suggest that the youth athletes tend to be aware of the nutrient requirements (6), however, this knowledge is often not translated into practice due to the limited convenience and peer pressure (7). Such programs, most often emphasize short-term performance outcomes while overlooking environmental impacts and the broader health implications of dietary choices (8). A strong preference for high-protein, resource-intensive convenience foods is commonly observed in team canteens and training camps, which, not only contribute to environmental degradation but also limits the inclusion of effective plant-based options (9). Although elite sports institutions have already started adopting greener policies, such as incorporating plant-based nutrition for post-training recovery and reducing plastic use, these efforts have not yet extended to youth sport (10). Integrating environmental metrics, such as carbon footprint reductions from plant-based diets, into sports nutrition research directly supports the dual goal of optimizing athletic performance and advancing environmental stewardship (11).

From a health perspective, sustainable nutrition emphasizes whole, plant-based, and minimally processed, which are associated with improved metabolic function, reduced risk of obesity, and enhanced long-term wellness (12). Prior studies have largely examined nutritional knowledge or healthy eating behaviors, whereas the integration of sustainability principles into youth sports nutrition remains largely unexplored (13). A limited number of educational and behavioral programs have begun integrating sustainability into sports nutrition for athletes, demonstrating improved awareness and modest dietary changes when sustainability principles were incorporated into sports nutrition education (14). However, these efforts remain limited in scope and geographic coverage, highlighting the need for evidence-based approaches that simultaneously examine performance and ecological dimensions.

The objectives of this research are to examine the impact of sports nutrition on the participation, motivation, and performance of youth athletes. More specifically, the study investigates the effect of energy intake, recovery, and health in the physiological domain as well as motivation and environmental concern within the psychosocial domain.

## 2 Conceptual and theoretical framework

### 2.1 Sustainable sports nutrition

Sustainable sports nutrition focuses on dietary methods that enhance athletic performance and recovery while reducing environmental harm. In the context of youth athletes, sustainable sports nutrition aims to support growth, development, and performance while fostering awareness of food system stewardship and environmental responsibility (15).

### 2.2 Youth participation

Participation of youths in sport is a complex construct that includes the frequency and the duration of engagement in both structured and unstructured physical activities, along with the overall quality of the experience. Such engagement is influenced by multiple factors, including access to facilities, perceived competence, motivation forces, interpersonal interactions, and structural contexts. Particular attention should be given to adolescents, as the levels of disengagement during this development stage are high, in addition, behavioral patterns developed in adolescence are also strong predictors of lifelong physical activity participation (16).

### 2.3 Motivation

Sports motivation may be described in terms of intrinsic and extrinsic factors. Regarding youth athletes, intrinsic motivation, which includes enjoyment, mastery, and personal development is widely regarded as more beneficial for sustained long-term participation, whereas extrinsic motivation, expressed through recognition, achievements, and parental support, is generally considered a facilitative factor (17).

### 2.4 Environmental awareness

Environmental awareness refers to the extent to which young athletes recognize and understand the ecological impact of their diet and lifestyle behaviors. Education about sustainable foods c and nutrition can reinforce these values and responsibility which can extend towards non-sport related lifestyle activities (18).

### 2.5 Conceptual model

This study’s conceptual framework follows a single, sequential pathway derived from the theories outlined in the preceding section. This arrangement aligns with behavior-change models that position knowledge and awareness as antecedents of dietary practices (19). Specifically, sustainable nutrition practices (e.g., locally-sourced food, waste reduction, and plant-based diets, etc.) are proposed to contribute to health benefits (overall wellness, enhanced recovery, and increased energy), which in turn influence motivation (self-efficacy, competence, intrinsic and extrinsic motivators). These motivational factors are expected to support youth participation through increased engagement, sustained involvement, reduced dropout, while environmental awareness functions as a moderating factor that reinforces motivation and participation, as shown in Table 1.

**Table 1:** Description of theoretical constructs operational definitions and measurement indicators.

Variable/concept	Definition	Theoretical basis	Expected relationship
Sustainable sports nutrition	Performance-supportive and eco-conscious dietary practices	SCT, BCT	Direct positive effect on health outcomes and awareness
Health benefits	Improved energy, recovery, and general well-being from dietary practices	SDT (competence), SCT (outcome expectancies)	Mediates the relationship between nutrition and motivation
Motivation	Intrinsic and extrinsic drivers of continued engagement in sport	SDT (autonomy, competence, relatedness)	Increases likelihood of sustained youth participation
Youth participation	Frequency, duration, and quality of sport involvement	SDT, SCT	Final outcome variable, positively influenced by motivation and health
Environmental Awareness	Recognition of ecological impacts of food choices	SCT (social modelling), BCT (education, support)	Moderates and strengthens the link between nutrition and participation

**Figure 1:**
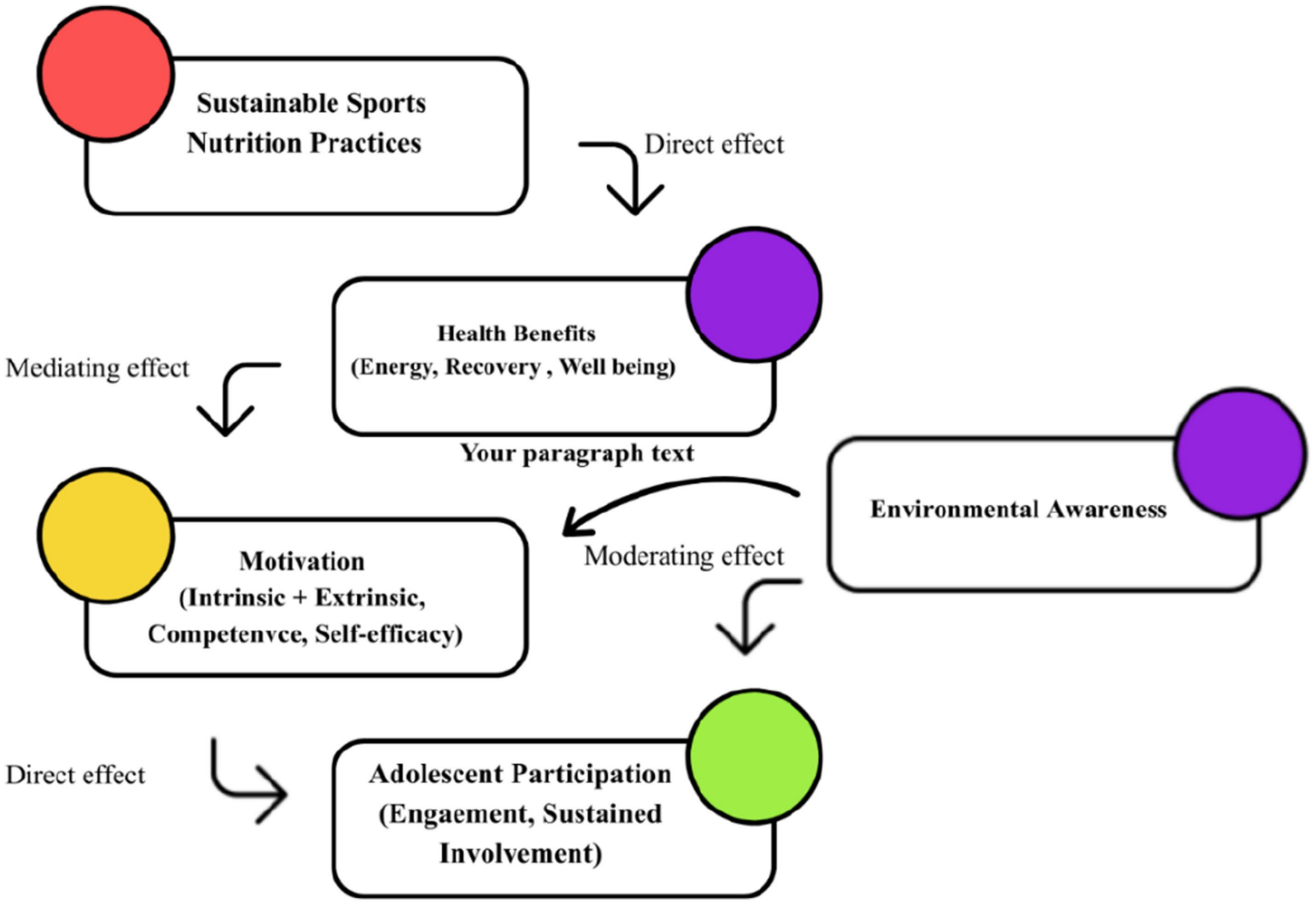
Conceptual model of hypothesized relationships among sports nutrition knowledge SNKQ, environmental awareness, and sustainable dietary behavior.

The conceptual model presented in Figure 1 explicitly illustrates the hypothesized pathways tested in the present analysis: SNKQ → Environmental awareness → sustainable dietary behavior, including both direct and moderate pathways (knowledge → behavior, awareness as mediator/ moderator).

### 2.6 Theoretical integration and application

The integration of Self-Determination Theory (SDT) (20), Social Cognitive Theory (SCT) (21), and Behavior Change Theory (BCT) (22) provides a coherent, multilevel framework for examining sustainable nutrition behaviors among adolescent athletes. SDT explains the internal motivations that drive behavior (autonomy, competence, relatedness), whereas SCT emphasizes the social and environmental conditions that influence modelling and reinforcement. BCT complements these frameworks by identifying practical mechanisms such as goal setting, feedback and reinforcement that facilitates behavior adoption (23).

## 3 Methodology

### 3.1 Study design, population and sample

**Figure 2:**
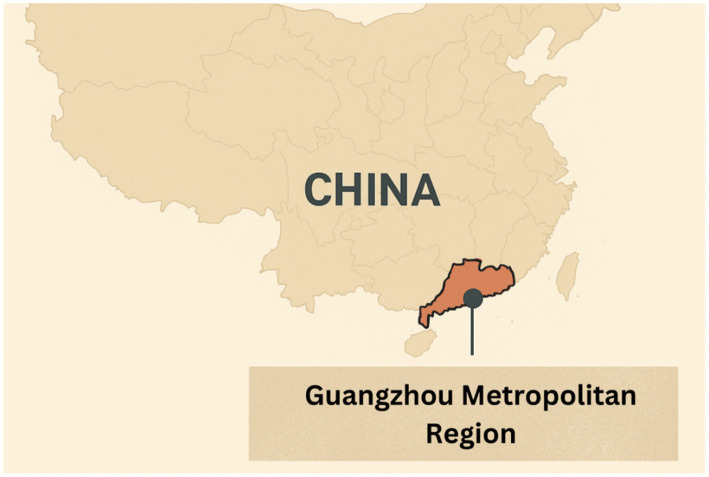
Guangzhou metropolitan region, China.

This study used a cross-sectional, observational design to examine associations between sports nutrition knowledge, environmental awareness, and self-reported sustainable nutrition practices among adolescent athletes (24). A stratified sampling approach was used to recruit the participants and ensure representation across key strata relevant to adolescent athletes, including sport modality (team vs. individual), competitive level (regional vs. national), and age group (13–15 vs. 16–18 years) (25). The sampling frame consisted of registered athletes associated with sports clubs and academies within the Guangzhou metropolitan area of China, as shown in Figure 2.

A total of 312 athletes who met the eligibility criteria were invited to participate in the study across 32 sports clubs. The total response rate was 38.5% (120/312). Among the cohort of athletes who initially enrolled, the completion rate was 80% (120/150). The a-priori power analysis conducted using G*Power assumed a medium effect size (r = 0.30), *α* = 0.05, and power = 0.80, yielding a required sample size *n* ≈ 85 for simple correlational analyses. The final sample of *n* = 120 therefore provides sufficient power for primary correlational and regression analyses.

### 3.2 Data collection methods

#### 3.2.1 Quantitative data

Data were collected using a structured questionnaire divided into three sections: (1) demographic characteristics and sports participation profiles, (2) nutrition knowledge and dietary practices, and (3) motivational and environmental awareness factors. The Sports Nutrition Knowledge Questionnaire (SNKQ) was adapted to include items reflecting knowledge of, and engagement, in sustainable dietary practices, e.g., selection of plant-based protein sources and locally sourced food (26).

#### 3.2.2 Operationalization of sustainable practices

To assess weather participants dietary practices were sustainable for research purposes, a transparent and reproducible composite Sustainable Practice Score (SPS), ranging from 0 to 10, was developed based on four observable, self-reported behaviors collected through the survey (27). The composite SPS demonstrated satisfactory internal consistency (Cronbach’s *α* = 0.81) and significant positive correlations with environmental awareness (r = 0.46, *p* < 0.001) and sports nutrition knowledge (r = 0.39, *p* < 0.001), indicating convergent validity. An inverse correlation with the meat-serving proxy (r = −0.31, *p* = 0.002), further supported criterion validity (28).

#### 3.2.3 Qualitative data

For the purpose of this study, semi-structured interviews were conducted with 50 participants, including 30 athletes, 10 coaches, 10 parents. The interviews were focused on participants’ perceptions of sustainable nutrition, observed behavioral changes, and perceived barriers and facilitators to adoption within the context of youth sports.

### 3.3 Variables and indicators

The study framework was used to structure the variables for the study. The variables and corresponding measurement tools used in the study are presented in Table 2.

**Table 2:** Variables, indicators, and measurement tools.

Variable	Indicator(s)	Measurement tool
Sustainable nutrition practices	Plant-based intake, local sourcing, waste reduction	Adapted Sport Nutrition Knowledge Questionnaire (SNKQ)
Health & performance benefits	Energy levels, recovery time, well-being	Self-report scale, observational data
Motivation	Intrinsic and extrinsic motivation levels	Motivation Scale for Youth Sports (MSYS)
Youth participation	Frequency of training, competitions, dropout intent	Structured survey, attendance logs
Environmental awareness	Perception of ecological impact of diet	Awareness subscale, qualitative interviews

### 3.4 Data analysis techniques

Quantitative data were analyzed using SPSS version 27 (29). Pearson’s correlation coefficients were calculated to examine associations between sustainable nutrition practices and participation-related variables, while multiple regression analyses were conducted to test predictive relationships. Moderation analyses were performed using the PROCESS macro to assess the extent to which environmental awareness influenced the nutrition–participation relationship.

Prior to analysis, data were screened for missing values, outliers and normality and all statistical assumptions were verified (30).

Effect sizes were reported as Cohen’s *r* and standardized *β* coefficients, where applicable. To control for multiple-comparison, the Benjamini-Hochberg false discovery rate (FDR) procedure was applied at q = 0.05. Assumptions for parametric testing including normality, linearity, multicollinearity, and homoscedasticity were examined and met prior to correlation and regression analyses (31).

Quantitative analysis was analyzed using the six-steps thematic analyses framework proposed by Braun and Clarke. Regression models were used to examine predictors of youth participation and motivation (32). The overall regression model predicting motivation was statistically significant (*F* (4, 115) = 8.64, *p* < 0.001, R^2^ = 0.26, adjusted R^2^ = 0.24), with sustainable nutrition emerging as a significant predictor (*β* = 0.32, *p* < 0.01) (33). Variance inflation factors (VIFs) values ranged from 1.12–1.38, indicating no multicollinearity concerns.

## 4 Results

### 4.1 Descriptive statistics and participants characteristics

The data obtained from 120 youth athletes (mean age = 15.2 years, SD = 1.8; 54% male, 46% female) indicated a strong association between nutrition practices and their impact on the individual’s health and participation in sports activities. The results showed that 78% of the participants were indicated as moderately aware of the environmental implications of their eating habits. In addition, 62% of the respondents reported adopting at least one sustainable dietary action, such as reducing meat consumption or incorporating locally grown foods into their diets.

Baseline dietary habits revealed that 68% of participants reported consuming meat more than four times per week, 41% reported regular consumption of plant-based meals, and 27% reported actively consuming locally sourced food options prior to participation in the study. A summary of these descriptive statistics is presented in Table 3.

**Table 3:** Participants demographics, sport profiles, and baseline dietary practices (*n* = 120).

Variable	Category/metric	n (%) or mean ± SD
Age (years)	-	15.2 ± 1.8
Gender	Male/female	65 (54.1%)/55 (45.8%)
Sport type	Team/individual	60%/40%
Weekly training hours	-	8.3 ± 3.2
Competition level	Regional/national	48%/52%
Plant-based meals ≥ 4x/week	-	41%
Meat consumption ≥ 3x/week	-	68%
Local food sourcing	-	27%

Mean sustainable practices score (SPS) was 5.64 ± 1.32, indicating moderate adherence to sustainable dietary behaviors (34). Using the SPS (possible range 0–10) as described in Methods section, scores ranged from 2.1–8.8. Category-based distribution showed that 22 participants (18.3%) were classified as low adherence (<3.5), 63 (52.5%) as moderate adherence (3.5–6.4), and 35 (29.2%) as high adherence (≥ 6.5). The SPS demonstrated acceptable internal consistency (Cronbach’s *α* = 0.81).

Correlation analyses indicated that SPS was positively associated with environmental awareness (r = 0.46, *p* < 0.001) and sport nutrition knowledge (SNKQ; r = 0.39, *p* < 0.001). Sensitivity analysis using weekly meat servings as a proxy for dietary carbon-footprint revealed a negative but weaker association (r = −0.31, *p* = 0.002), supporting construct validity.

**Figure 3:**
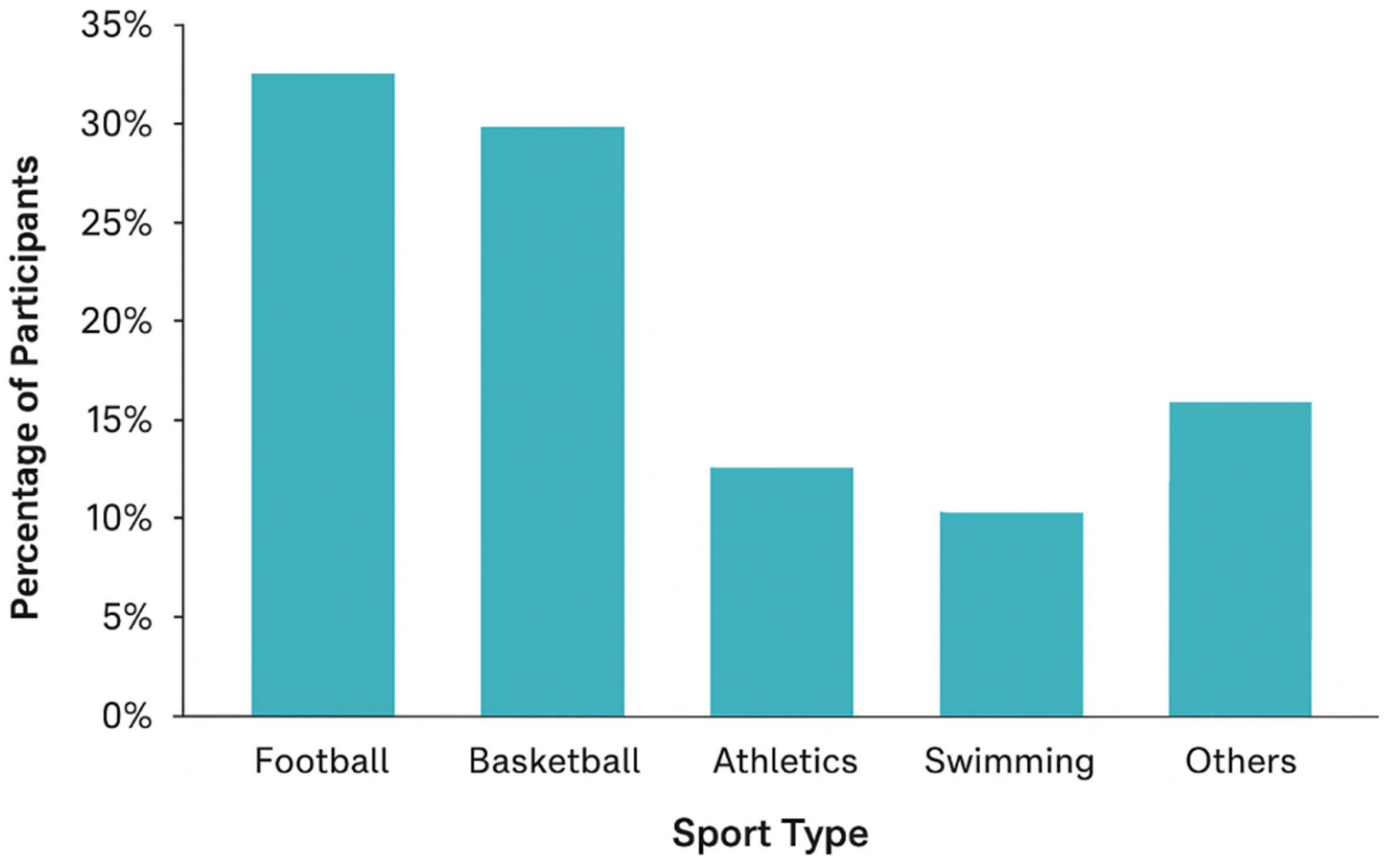
Percentage of participants stratified by sport type.

Participants represented five primary sport categories; football (32%), basketball (30%), badminton (21%), athletics (12%), swimming (10%), and other sports (15%) as presented in Table 4 and Figure 3.

**Table 4:** Demographic characteristics of adolescent athletes, including sport type.

Sport type	*n* (%)	Male (%)	Female (%)	Mean training hours/week (SD)
Football	32%	70%	30%	6.2 (±1.4)
Basketball	30%	60%	40%	5.1 (±1.8)
Badminton	21%	50%	50%	6.5 (±1.2)
Athletics	12%	55%	45%	7.0 (±2.1)
Swimming	10%	58%	42%	6.8 (±1.5)
Others	15%	40%	60%	4.3 (±1.1)

### 4.2 Quantitative analysis

Key quantity fundings are summarized in Tables 5, 6. Sustainable nutrition practices were positively associated with motivation (r = 0.46, *p* < 0.01) and participation frequency (r = 0.39, *p* < 0.01). Regression analyses indicated that nutrition knowledge and environmental awareness were significant predictors of sustainable dietary behavior, independent of demographic variables.

**Table 5:** Pearson correlation coefficient among key variables with significance values.

Variable pair	*n*	r/β	*p*	Interpretation
Sustainable nutrition – motivation	120	r = 0.46	< 0.01	Strong positive association
Sustainable nutrition – participation	120	r = 0.39	< 0.01	Moderate positive association
Environmental awareness × nutrition → motivation	120	β = 0.27	< 0.05	Awareness strengthens the nutrition
Plant-based diet frequency – recovery time	120	r = − 0.34	< 0.01	More plant-based meals associated with faster recovery

**Table 6:** Pearson correlation coefficients and multiple linear regression analysis.

Predictor variable	Outcome variable	r	β (standardized)	SE	t	*p*	Effect size (ɳ^2^/d)
SNKQ score	Sustainable diet adoption	0.39	0.34	0.08	4.21	<0.001	ɳ^2^ = 0.12
Environmental awareness	Sustainable diet adoption	0.46	0.41	0.07	4.96	<0.001	ɳ^2^ = 0.17
Age	Sustainable diet adoption	0.12	0.09	0.06	1.55	0.124	-

To further identify independent predictors, multiple regression models were computed using sustainable diet adoption as the outcome variable (Table 6). Sports nutrition knowledge (*β* = 0.34, *p* < 0.001) and environmental awareness (β = 0.41, *p* < 0.001) remained significant predictors after controlling for age and competitive level (35). These variables explained approximately 29% of the variance in sustainable dietary behavior (adjusted R2 = 0.29). No multicollinearity of regression assumptions was detected (VIF < 2; Levene’s tests, *p* > 0.05).

**Figure 4:**
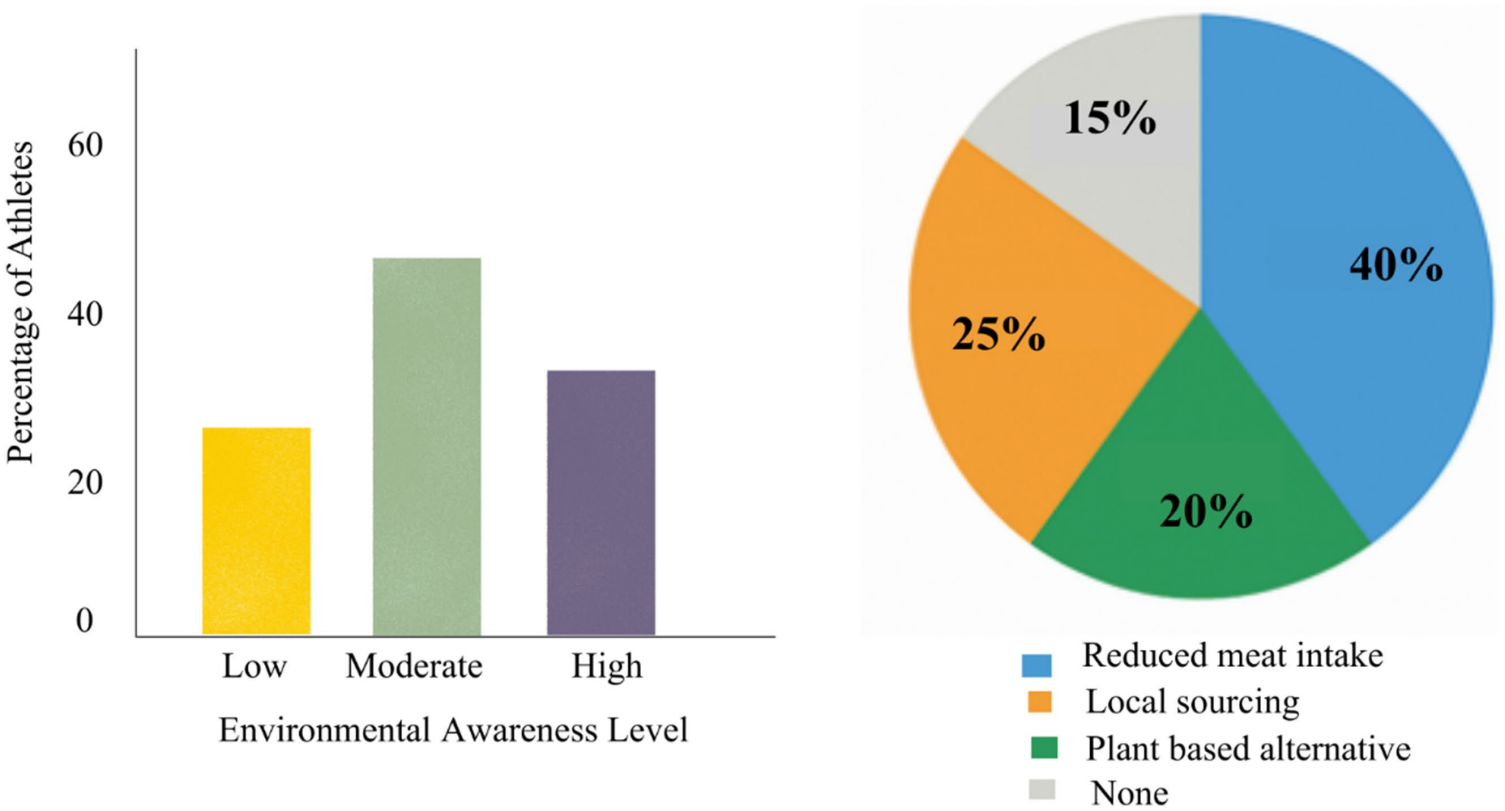
Awareness and adoption of sustainable nutrition practices.

Figure 4 illustrates the distribution of environmental awareness levels and demonstrates how these categories translated into sustainable dietary adoption. Athletes with higher levels of environmental awareness were significantly more likely to adopt at least one sustainable nutrition practices (X^2^ (*n* = 120) = 9.34, *p* < 0.1), supporting the moderating role of environmental awareness proposed in the conceptual framework.

Youth athletes are strongly influenced by parents, coaches, and team members. Participants who adopted locally sourced and plant-based alternative predominantly reported parental and team support as the motivating forces, consistent with SCT, which posits that behaviors are learned and reinforced within social contexts (36).

BCT provides further insights into adoption patterns with respect to the intention-behavior gap. Although 40% of the respondents reducing meat consumption, only 20% of these individuals also transitioned to regular consumption of plant-based alternatives. This discrepancy may reflect perceived barriers, including cost concern regarding inadequate protein intake.

### 4.3 Qualitative findings

From a sport involvement perspective, the findings support the hypothesis that nutrition plays an important role in youth participation in sport (37). The observed correlations between sustainable dietary behaviors, motivational variables, and perceived performance indicate that positive outcomes for athletic participation can be achieved among youth athletes.

The present findings align with prior research demonstrating that higher levels of sport nutrition knowledge and environmental awareness are associated with more sustainable dietary behaviors (38, 39). The observed effect sizes (r ≈ 0.34–0.46) are relatively strong for adolescent sport populations, suggesting that the combination of nutritional knowledge and environmental concern meaningfully enhance behavior change.

### 4.4 Mixed-methods integration

Integration of the quantitative and qualitative findings revealed convergence between the measured associations and participants narratives. This convergence reinforces the interpretation that motivational and social-cognitive factors jointly underpin the adoption of sustainable nutrition practices among adolescent athletes (40).

## 5 Discussion

The findings are consistent with existing literature highlighting the importance of nutrition education and environmental awareness in shaping sustainable eating behaviors among adolescents athletes (41). Previous studies have shown that higher sport nutrition literacy is associated with healthier dietary patterns and greater participation in sport activities (42). The present study further demonstrated that sustainability-oriented dietary behaviors are influenced not only by cognitive factors but also by motivational constructs and level of sports participation.

The combined results of the mixed-method study provided a consistent, multidimensional understanding of how sustainability-oriented nutritional behaviors evolve within youth sports settings, and how these practices are connected to physiological outcomes and psychosocial engagement, in line with findings from other mixed-methods research (43).

The quantitative findings indicated a positive, moderated relationship between motivation and sport participation and sustainable nutrition practises was moderate. These association remained significant after accounting for demographic factors, suggesting the sustainable dietary beheviors can be positively correlated with adolescent engagement in sports (44). The predictive roles of sport nutrition knowledge and environmental awareness further underscores the importance of cognitive and attitudinal elements in behavioral development (45). Adolescents who demonstrated a better understanding of the nutritional principles and an increased awareness of the ecological consequences were more likely to adopt sustainable dietary behaviors, highlighting the role of education during this formative life stage (46).

The qualitative data contributed to these observations by elucidating how athletes interpret sustainable nutrition in relation to their training experience. A significant number of the athletes reported improvements in energy level, and recovery and overall well-being, providing experimental evidence for the positive association between sustainable dietary practises and motivation identified in the qualitative analyses, and consistent with previous research in adolescent performance nutrition (47). Consistent with Social Cognitive Theory, and Self-Determination Theory, the qualitative findings highlighted parental and coach modelling as key facilitators of sustainable dietary adoption (48).

From a theoretical perspective, the current results are consistent with Self-Determination Theory (49), which is based on the notion that the internalization of competence and value-based principles supports sustained behavioral engagement. When athletes perceive sustainable nutrition practices as contributing to performance enhancement and reduced ecological impact, the chances of internalization increases, thereby strengthening intrinsic motivation. Similar motivational processes have been observed in studies of youth physical activity and health behavior, in which value-congruent practices promote persistence and long-term engagement (50).

The statistical findings also depicted a steady intention-behavior gap. Although a substantial percentage of the participants showed awareness of sustainable practices, only a subset reported regular implementation. Qualitative evidences identified several structural barriers, including financial limitations, limited access to plant-based foods, inadequate cooking skills, and misinformation regarding protein adequacy (51). These obstacles provide practical explanations for variation in adoption levels, and support the importance of favorable conditions, consistent with the core principles of Behavioral Change Theory. Notably, increased awareness can be insufficient to translate into sustained practices when adolescents lacked environmental support or reinforcement.

The integration of quantitative and qualitative data highlights two complementary approaches through which sustainable nutrition may support long-term involvement in youth sports programs. Psychologically, dietary patterns that emphasize to plant-based and minimally processing foods may promote faster recovery and keep an individual at a stable energy level, which in turn encourages better consistency in training (52). Physiologically, the adoption of environmentally sustainable dietary practices may foster alignment with personal and environmental values, which in turn enhances intrinsic motivation. Collectively, these mechanisms demonstrated the value of a mixed-method approach, as it not only documents measurable associations, but also elucidates the experimental processes that shape sustainable nutrition behavior among adolescents.

These findings have practical implications for youth sport programs, schools, and families. Youth sport programs, schools and families hold strategic roles in influencing dietary behaviors, these settings can increase sustainable nutrition practices by increasing the availability of plant-based foods, reducing reliance on highly processed food and minimizing single-use packaging (53). On a larger scale, the incorporation of the sustainability concepts into institutional policies and nutritional guidelines may contribute to the development of long-term, health promoting behaviors during adolescence, a particularly formative stage for establishing lifelong behaviors (54).

Although most previous studies have focused on either sport nutrition behaviors or sustainability related dietary choices (28), the present study extends the literature by empirically integrating these two domains within the context of youth sport (55). The effect sizes observed were comparable to, and in some cases larger than, those reported in previous studies on adolescent dietary behavior, suggesting that sport settings may represent particularly promising contexts for fostering sustainability-oriented nutrition practices (56).

### 5.1 Limitations

This study has some limitations. First, dietary behaviors, environmental awareness, motivation, and recovery performance were assessed using self-report tools, which are inherently susceptible to recall bias and social desirability effects. Second, the sample was surveyed from a single metropolitan area in China, limiting the external validity of the findings to the adolescents living in rural or different cultural areas or sport systems that may vary in terms of the availability of resources. Finally, although the SPS demonstrated acceptable internal consistency and initial evidence of construct validity, it remains a proxy measure and does not directly measure environmental impact.

## 6 Conclusion and future recommendations

The current study analyzed the effects of the implementation of sustainable sports nutrition practices on participation, motivation, performance, and environmental awareness among youth athletes. These findings suggests that sustainable nutrition practices support both sport engagement and environmental awareness in adolescent athletes. Specifically, environmentally responsible dietary attitudes were associated with positive physiological perceptions and higher level of psychosocial activity among young athletes. Sustainable nutrition practices were positively correlated with motivation (r = 0.46, *p* < 0.01) and participation frequency (r = 0.39, *p* < 0.01), with environmental awareness moderating both relationships. The qualitative findings corroborated these results, indicating that athletes perceived greater energy level, faster recovery, and increased engagement with sustainable nutrition practices. Collectively, these findings indicate that awareness alone is insufficient to drive sustained adoption of sustainable dietary behaviors. Adoption appears to depend on social modelling, parental support, and structural support within schools and sports clubs. Future research should benefit from longitudinal or multi-wave study design to determine how sustainable dietary behaviors develop and to examine their relationship with objective performance outcomes. Additionally, cross-cultural or multi-regional comparisons are also necessary to explore how variations in food environments and sport systems influence the knowledge-to-behavior pathway.

## Data Availability

The raw data supporting the conclusions of this article will be made available by the authors, without undue reservation.
